# Autoimmune Hypophysitis: A Cause of Central Diabetes Insipidus

**DOI:** 10.7759/cureus.80369

**Published:** 2025-03-10

**Authors:** Debashis Priyadarshan Sahoo, Bikash R Rout, Gwenette War

**Affiliations:** 1 Department of Internal Medicine, All India Institute of Medical Sciences, Guwahati, Guwahati, IND; 2 Department of General Medicine, Barasat Government Medical College, Kolkata, IND; 3 Department of General Medicine, All India Institute of Medical Sciences, Guwahati, Guwahati, IND

**Keywords:** adrenal suppression, antidiuretic hormone, autoimmune hypophysitis, diabetes insipidus, polydipsia and polyuria

## Abstract

Diabetes insipidus (DI) is a rare disorder characterized by polyuria and polydipsia due to a deficiency of antidiuretic hormone (ADH) or insensitivity to ADH. This case report presents a rare cause of DI secondary to autoimmune hypophysitis, emphasizing the diagnostic and management challenges associated with the condition. The discussion highlights pathophysiology, clinical presentation, diagnostic approaches, and therapeutic strategies. The importance of considering autoimmune hypophysitis in the differential diagnosis of central DI is underscored, along with long-term implications of the disease.

## Introduction

Diabetes insipidus (DI) is a disorder of water balance caused by either inadequate secretion of antidiuretic hormone (ADH), i.e., central DI or renal resistance to ADH (nephrogenic DI). Central DI arises due to damage to the hypothalamic-pituitary axis, which can occur due to trauma, tumors, infiltrative diseases, infections, or autoimmune conditions [1]. The disorder results in excessive water loss in urine, leading to hypernatremia, dehydration, and compensatory polydipsia [2].

Autoimmune hypophysitis is an increasingly recognized cause of central DI. It involves lymphocytic infiltration of the pituitary gland, resulting in pituitary dysfunction and, in some cases, isolated or combined hormone deficiencies [3]. The disease is often associated with other autoimmune disorders, including Hashimoto’s thyroiditis, Addison’s disease, and type 1 diabetes mellitus [4]. The condition primarily affects women, particularly during pregnancy or the postpartum period, but can also occur in men and nonpregnant women [5].

Recognizing autoimmune hypophysitis as a cause of central DI is essential, as early intervention can prevent irreversible pituitary damage. This case report discusses a rare presentation of DI secondary to autoimmune hypophysitis and highlights diagnostic and therapeutic challenges.

## Case presentation

A 35-year-old woman presented to the outpatient endocrinology clinic with a four-month history of progressively worsening symptoms, including excessive thirst (polydipsia), increased urinary frequency (polyuria), and nocturia. She described an intense and persistent urge to drink fluids, estimating a daily water intake of approximately 8 to 10 liters, far exceeding normal hydration needs. She also noted that she was waking up 3 to 4 times per night to urinate, with large volumes of dilute urine, which significantly disrupted her sleep. In addition to these primary complaints, the patient reported a constellation of secondary symptoms, including generalized fatigue, intermittent episodes of dizziness (particularly upon standing), and mild, non-specific headaches occurring several times per week. She denied any associated nausea, vomiting, or visual disturbances that might suggest increased intracranial pressure.

The patient’s history was carefully explored to identify potential etiologies. She explicitly denied any history of head trauma, recent or remote intracranial infections (e.g., meningitis or encephalitis), radiation therapy to the head or neck, or exposure to medications known to affect renal tubular function or pituitary regulation, such as lithium, demeclocycline, or other nephrotoxic agents. Furthermore, she reported no symptoms suggestive of adrenal insufficiency (e.g., salt craving, hyperpigmentation, or orthostatic hypotension beyond mild dizziness), thyroid dysfunction (e.g., heat/cold intolerance, palpitations, or significant weight changes), or hypercalcemia (e.g., constipation, bone pain, or confusion). Her review of systems was otherwise unremarkable, with no fever, night sweats, or unintentional weight loss to suggest malignancy or chronic infection.

Her past medical history was notably benign. She had no prior diagnoses of chronic illnesses such as hypertension, diabetes mellitus, or renal disease, and she had never undergone surgery. Her menstrual cycles remained regular, with a consistent 28- to 30-day cycle and no recent changes in flow or associated symptoms, ruling out overt hypothalamic-pituitary-ovarian axis dysfunction. She was not taking any regular medications, including over-the-counter supplements, and denied recreational drug use or excessive alcohol consumption. A detailed family history was obtained, revealing no evidence of endocrinopathies (e.g., DI, pituitary tumors, or hypopituitarism) or autoimmune disorders (e.g., polyglandular syndromes or systemic lupus erythematosus) among first- or second-degree relatives. Socially, she was a non-smoker, employed as an office administrator, and lived with her partner in an urban setting, with no significant environmental exposures reported.

Physical examination

On physical examination, the patient appeared clinically well-hydrated, with no overt signs of dehydration such as dry mucous membranes, sunken eyes, or reduced skin turgor. Her general appearance was that of a healthy adult woman with a body mass index (BMI) within the normal range (approximately 22 kg/m², based on observed physique), and she was alert, oriented, and cooperative throughout the assessment. Her vital signs were recorded as follows: blood pressure was 116/76 mmHg, measured in the seated position using an automated cuff on the right arm, indicating normotension without evidence of orthostatic changes when repeated standing (no significant drop noted); the heart rate was 78 beats per minute (bpm), regular and of normal volume, assessed via radial pulse; oral temperature was 36.8°C, within the normal range and excluding fever; and the respiratory rate was 16 breaths per minute, with an unlabored breathing pattern and no use of accessory muscles.

A comprehensive neurological examination was performed and found to be entirely unremarkable. Cranial nerve testing revealed intact function, with normal visual acuity, pupillary light reflexes (both direct and consensual), and extraocular movements. Motor strength was 5/5 in all extremities, with normal tone, reflexes (2+ and symmetric at biceps, triceps, knees, and ankles), and coordination (finger-to-nose and heel-to-shin testing intact). Sensory examination showed no deficits to light touch, pinprick, or vibration. Gait was steady, and there were no signs of cerebellar dysfunction or tremor. A fundoscopic examination was conducted using an ophthalmoscope, revealing sharp disc margins bilaterally, with no evidence of papilledema, optic atrophy, or retinal hemorrhages, effectively ruling out raised intracranial pressure as a contributing factor to her headaches or other symptoms.

Examination of the skin and mucous membranes demonstrated no abnormalities. There was no hyperpigmentation suggestive of adrenal insufficiency (e.g., darkening of creases, scars, or buccal mucosa), nor were there hypopigmented patches or vitiligo to indicate autoimmune processes. The oral cavity and lips were moist and without lesions, and no mucosal abnormalities were observed. The thyroid gland was palpated and found to be of normal size, smooth, and non-tender, with no palpable nodules or goiter, and no associated bruit on auscultation, making overt thyroid pathology less likely. The neck examination also confirmed the absence of cervical lymphadenopathy.

A systemic examination was conducted to exclude multisystem involvement. There was no palpable lymphadenopathy in the axillary, inguinal, or supraclavicular regions, reducing suspicion of malignancy or chronic infection. The cardiovascular examination revealed normal heart sounds (S1 and S2) without murmurs, gallops, or rubs, and peripheral pulses were intact. Respiratory examination showed clear lung fields bilaterally with normal vesicular breath sounds and no wheezing, crackles, or dullness to percussion. The abdomen was soft, non-tender, and non-distended, with no palpable hepatosplenomegaly or masses; bowel sounds were present and normoactive. Extremities showed no edema, clubbing, or cyanosis, and joints were without swelling or deformity.

Provisional diagnoses

Given her symptoms, the primary differential diagnoses included: (i) Central DI (due to autoimmune hypophysitis, neoplasms, trauma, infections, or genetic causes); (ii) Nephrogenic DI (due to renal insensitivity to ADH); (iii) Primary polydipsia (excessive water intake due to psychological disorders or hypothalamic dysfunction); (iv) Hypercalcemia or hypokalemia-induced polyuria; (v) Osmotic diuresis (due to uncontrolled diabetes mellitus or hyperglycemia).

Investigations

The patient's investigative findings are summarized across four tables, providing a comprehensive diagnostic profile. Table 1 presents the basic metabolic and electrolyte panel, highlighting mild hypernatremia, elevated serum osmolality, and inappropriately dilute urine, consistent with DI. Table 2 details the endocrine and pituitary function tests, revealing central DI, secondary adrenal insufficiency, and subclinical hypothyroidism, with hormonal patterns suggestive of underlying autoimmune hypophysitis. Table 3 outlines the water deprivation test and desmopressin challenge, confirming central DI through a lack of urine concentration in response to dehydration and a significant rise in urine osmolality after desmopressin administration. Table 4 includes autoimmune and inflammatory markers along with additional investigations, demonstrating positive anti-pituitary antibodies indicative of autoimmune hypophysitis. These findings collectively support a unifying diagnosis of autoimmune hypophysitis with multiple pituitary hormone deficiencies.

**Table 1:** Basic Metabolic and Electrolyte Panel BUN: Blood urea nitrogen; DI: Diabetes insipidus; Na⁺: Sodium; K⁺: Potassium; Cl⁻: Chloride; HCO₃⁻: Bicarbonate; mg/dL: Milligrams per deciliter; mmol/L: Millimoles per liter; mOsm/kg: Milliosmoles per kilogram.

Test	Result	Reference Range	Interpretation
Serum sodium	148 mmol/L	135–145 mmol/L	Mild hypernatremia due to free water loss, consistent with diabetes insipidus.
Serum potassium	4.1 mmol/L	3.5–5.0 mmol/L	Normal, which excludes hypokalemia as a cause of nephrogenic DI.
Serum chloride	108 mmol/L	98–106 mmol/L	Slightly elevated, likely secondary to hypernatremia and water deficit.
Serum bicarbonate	24 mmol/L	22–29 mmol/L	Normal, no metabolic acidosis or alkalosis.
Blood urea nitrogen (BUN)	12 mg/dL	7–20 mg/dL	Normal, which suggests adequate renal perfusion despite polyuria.
Serum creatinine	0.8 mg/dL	0.6–1.2 mg/dL	Normal, which indicates intact renal function; polyuria not due to renal failure.
Blood glucose	89 mg/dL	70–100 mg/dL	Normal, which rules out hyperglycemia-induced osmotic diuresis.
Serum calcium	9.3 mg/dL	8.5–10.5 mg/dL	Normal, which excludes hypercalcemia as a cause of nephrogenic DI.
Serum magnesium	2.0 mg/dL	1.7–2.2 mg/dL	Normal, no magnesium-related tubular dysfunction.
Serum osmolality	305 mOsm/kg	275–295 mOsm/kg	Elevated, which reflects hyperosmolar state from water loss in DI.
Urine osmolality	120 mOsm/kg	>600 mOsm/kg (with hyperosmolality)	Inappropriately low, which indicates inability to concentrate urine, consistent with DI.
24-hour urine output	9 liters	<3 liters	Excessive, diagnostic of polyuria; supports DI diagnosis.

**Table 2:** Hormonal and Pituitary Function Tests ACTH: Adrenocorticotropic hormone; ADH: Antidiuretic hormone; DI: Diabetes insipidus; FSH: Follicle-stimulating hormone; GH: Growth hormone; IGF-1: Insulin-like growth factor-1; LH: Luteinizing hormone; T4: Thyroxine; TSH: Thyroid-stimulating hormone.

Test	Result	Reference Range	Interpretation
Plasma ADH levels	0.3 pg/mL	1–5 pg/mL	Inappropriately low for serum osmolality, diagnostic of central DI.
Thyroid function tests			
- Free T4	1.2 ng/dL	0.8–2.0 ng/dL	Normal, preserved thyroid hormone production in subclinical hypothyroidism.
- TSH	5.8 mIU/L	0.4–4.5 mIU/L	Mildly elevated, consistent with subclinical hypothyroidism; possible early pituitary involvement.
Morning cortisol (8 AM)	3.5 mcg/dL	5–25 mcg/dL	Low, suggestive of adrenal insufficiency.
ACTH levels	8.2 pg/mL	10–50 pg/mL	Low-normal, inappropriate for low cortisol, indicates secondary adrenal insufficiency.
ACTH stimulation test	Peak cortisol 12 mcg/dL	>18 mcg/dL at 60 min	Suboptimal response, which confirms adrenal insufficiency (likely pituitary origin).
Growth hormone (GH)	0.5 ng/mL	0.4–10 ng/mL (basal)	Low-normal, possible early GH deficiency; requires stimulation test for confirmation.
IGF-1	110 ng/mL	115–307 ng/mL (age-adjusted)	Slightly low, which supports possible GH axis impairment.
Prolactin	18 ng/mL	2–29 ng/mL (non-pregnant female)	Normal, no hyperprolactinemia to suggest pituitary mass effect.
LH	4.2 mIU/mL	2–12 mIU/mL (follicular phase)	Normal, consistent with regular menses; no gonadotropin deficiency.
FSH	5.1 mIU/mL	3–12 mIU/mL (follicular phase)	Normal, intact hypothalamic-pituitary-ovarian axis at this stage.

**Table 3:** Autoimmune and Inflammatory Markers ACE: Angiotensin-converting enzyme; ACTH: Adrenocorticotropic hormone; ANA: Antinuclear antibody; CRP: C-reactive protein; CSF: Cerebrospinal fluid; DI: Diabetes insipidus; ESR: Erythrocyte sedimentation rate; IgG4: Immunoglobulin G4; MRI: Magnetic resonance imaging; RF: Rheumatoid factor; TPO: Thyroid peroxidase; SLE: Systemic lupus erythematosus.

Test	Result	Reference Range	Interpretation
Anti-pituitary antibodies	Positive	Negative	Suggests autoimmune hypophysitis as a unifying etiology.
Anti-thyroid peroxidase (TPO) antibodies	Negative	Negative	No evidence of autoimmune thyroiditis.
Anti-adrenal antibodies	Negative	Negative	Rules out primary adrenal insufficiency (Addison’s disease).
Erythrocyte sedimentation rate (ESR)	25 mm/hr	0–20 mm/hr	Mildly elevated, possible low-grade inflammation.
C-reactive protein (CRP)	3.2 mg/L	<5 mg/L	Normal, no significant systemic inflammation.
ANA (antinuclear antibody)	Negative	Negative	No evidence of systemic autoimmune disease (e.g., SLE).
Rheumatoid factor (RF)	Negative	Negative	Excludes rheumatoid arthritis or related conditions.
Serum IgG4	45 mg/dL	4–86 mg/dL	Normal, less likely IgG4-related hypophysitis.

**Table 4:** Specialized Studies DI: Diabetes insipidus; mOsm/kg: Milliosmoles per kilogram; Na⁺: Sodium; UOsm: Urine osmolality; S.Osm: Serum osmolality.

Test	Result	Reference Range	Interpretation
Water deprivation test			
- Baseline serum osmolality	305 mOsm/kg	275–295 mOsm/kg	Elevated, confirming hyperosmolar state.
- Baseline urine osmolality	120 mOsm/kg	>600 mOsm/kg	Inappropriately low, suggesting ADH deficiency or resistance.
- Post-deprivation serum osmolality	310 mOsm/kg	275–295 mOsm/kg	Further elevation confirmed adequate stimulus for ADH release.
- Post-deprivation urine osmolality	120 mOsm/kg	>600 mOsm/kg	No increase, confirming DI.
- Post-desmopressin urine osmolality	650 mOsm/kg	>50% increase	Diagnostic of central DI (kidneys respond to ADH analog).
- Weight loss during test	2% (e.g., 60 kg to 58.8 kg)	<3–5% (safety limit)	Mild dehydration; test completed safely.
Desmopressin response	Urine osmolality 650 mOsm/kg	>50% increase post-desmopressin	Confirms central DI.
Visual field testing	Normal	Normal	No bitemporal hemianopia; no significant pituitary mass effect.
Chest X-ray	Normal	Normal	No evidence of sarcoidosis or tuberculosis affecting the pituitary.
Serum angiotensin-converting enzyme (ACE)	30 U/L	8–52 U/L	Normal, reduces likelihood of sarcoidosis.

Basic metabolic tests demonstrated mild hypernatremia and elevated serum osmolality, indicating a hyperosmolar state due to water loss. However, urine osmolality was inappropriately low, suggesting an impaired ability to concentrate urine. Given the normal blood glucose levels, osmotic diuresis secondary to hyperglycemia was excluded. Additionally, serum calcium and potassium were within normal limits, ruling out hypercalcemia- or hypokalemia-induced polyuria.

To differentiate between central DI, nephrogenic DI, and primary polydipsia, a water deprivation test was performed. The patient’s urine osmolality failed to increase despite rising serum osmolality, indicating that endogenous ADH was either deficient or ineffective. The subsequent administration of desmopressin (exogenous ADH) led to a marked increase in urine osmolality, confirming that the kidneys were responsive to ADH. This effectively ruled out nephrogenic DI and primary polydipsia, solidifying a diagnosis of central DI.

Given the presence of central DI, further investigation was conducted to identify an underlying cause, focusing on pituitary function and potential autoimmune, infiltrative, or neoplastic etiologies. Hormonal testing revealed secondary adrenal insufficiency, as evidenced by a low morning cortisol level with inappropriately low ACTH. An ACTH stimulation test confirmed a blunted adrenal response, indicating pituitary rather than primary adrenal dysfunction. Thyroid function tests showed subclinical hypothyroidism, likely central in origin, while IGF-1 levels were slightly low, raising suspicion for early GH deficiency. Normal gonadotropin levels suggested a preserved hypothalamic-pituitary-gonadal axis.

The presence of multiple pituitary hormone deficiencies raised concern for hypophysitis or another pituitary pathology. Autoimmune screening revealed positive anti-pituitary antibodies, strongly supporting autoimmune hypophysitis as the underlying cause. Other autoimmune markers, including anti-thyroid peroxidase and anti-adrenal antibodies, were negative, ruling out primary autoimmune thyroid or adrenal disease. Systemic autoimmune disorders such as systemic lupus erythematosus and rheumatoid arthritis were excluded based on negative ANA and rheumatoid factor results. Normal IgG4 levels made IgG4-related hypophysitis unlikely, and inflammatory markers were mildly elevated, suggesting a low-grade inflammatory process.

Structural imaging (MRI) was not performed, leaving the presence of a pituitary mass or infiltrative disease unconfirmed. However, the absence of visual field deficits suggested no significant compressive pituitary pathology. Likewise, CSF analysis was not conducted, as there were no clinical indications of CNS infections or infiltrative disorders.

By systematically excluding alternative diagnoses, the findings confirmed a final diagnosis of central DI, secondary adrenal insufficiency, and subclinical hypothyroidism due to autoimmune hypophysitis. The presence of positive anti-pituitary antibodies, along with evolving hypopituitarism, strongly suggests an autoimmune etiology, though continued endocrine follow-up and possible pituitary imaging would be necessary for further assessment.

Final diagnosis

Based on clinical and laboratory findings, a final diagnosis of central DI secondary to autoimmune hypophysitis was established, along with associated adrenal insufficiency and central hypothyroidism.

Treatment and outcome

The patient was started on desmopressin (DDAVP) 10 mcg intranasally twice daily. For adrenal insufficiency, she received hydrocortisone 20 mg in the morning and 10 mg in the evening. Levothyroxine 50 mcg daily was initiated for hypothyroidism. At a six-week follow-up, polyuria and polydipsia had resolved. Serum sodium levels normalized, and the patient reported significant improvement in fatigue and headaches. At three months, hormonal replacement therapy was well tolerated, with stable cortisol and thyroid function tests.

## Discussion

Autoimmune hypophysitis is a rare but relatively increasing cause of central DI. The autoimmune destruction of the pituitary gland leads to hormonal deficiencies, including ACTH, TSH, and ADH deficiencies, which were observed in our patient. The pathophysiology involves lymphocytic infiltration of the pituitary, leading to varying degrees of hypopituitarism. The rarity of this condition poses significant diagnostic and management challenges.

Several case reports and retrospective studies have described autoimmune hypophysitis as an underlying etiology of central DI. A review by Caturegli et al. reported that central DI was observed in nearly 25% of patients with autoimmune hypophysitis, typically manifesting later in the disease course [6]. Another study by Faje et al. highlighted that central DI in autoimmune hypophysitis is more commonly seen in postpartum women; however, cases in nonpregnant individuals, such as our patient, have been reported [7]. The distinction between idiopathic DI and autoimmune hypophysitis remains crucial, as the latter has long-term implications, including progressive pituitary insufficiency.

The differential diagnosis for central DI is broad, requiring careful evaluation. Neoplastic causes, such as craniopharyngioma or germinomas, typically present with other mass effects like headaches or visual disturbances. MRI was not performed in view of no strong evidence of compressive signs and symptoms; however, the presence of anti-pituitary antibodies strongly supported an autoimmune etiology over neoplastic causes. Granulomatous diseases like sarcoidosis and Langerhans cell histiocytosis may also present similarly but are usually accompanied by systemic symptoms or imaging findings [8].

Nephrogenic DI was effectively ruled out through the water deprivation test, which demonstrated a failure of urine osmolality to increase in response to rising plasma osmolality, confirming ADH deficiency rather than renal resistance. Furthermore, normal calcium and potassium levels excluded metabolic causes, and the absence of psychiatric symptoms ruled out primary polydipsia.

While autoimmune hypophysitis is well-documented in the literature, its manifestation as isolated DI with concurrent secondary adrenal insufficiency and central hypothyroidism is uncommon. Most cases of autoimmune hypophysitis present with anterior pituitary dysfunction first, with DI occurring later. Our case highlights an atypical presentation where DI was an early manifestation, emphasizing the need for heightened clinical suspicion in patients presenting with polyuria and polydipsia.

Patients with autoimmune hypophysitis require lifelong monitoring due to the risk of progressive pituitary hormone deficiencies. Studies suggest that up to 50% of cases may develop additional hormone deficits over time, necessitating ongoing endocrine assessment [9]. Our patient responded well to desmopressin, hydrocortisone, and levothyroxine therapy, achieving symptomatic relief and biochemical normalization at follow-up visits.

## Conclusions

This case highlights the critical importance of considering autoimmune hypophysitis as an underlying cause of central DI, especially in patients with multiple pituitary hormone deficiencies. While infiltrative and structural pituitary diseases are commonly evaluated, clinicians should maintain a high index of suspicion for autoimmune etiologies. Early recognition and timely initiation of appropriate hormonal replacement therapy are essential to prevent irreversible pituitary damage and associated endocrine complications. Moreover, this case underscores the necessity of a multidisciplinary approach in managing hypophysitis, as long-term follow-up is crucial for monitoring disease progression, optimizing therapy, and preventing secondary complications. Prompt diagnosis and proactive management of autoimmune-mediated pituitary dysfunction can significantly improve patient outcomes and quality of life.
